# Emergence and predominance of norovirus GII.17 in Huzhou, China, 2014–2015

**DOI:** 10.1186/s12985-015-0370-9

**Published:** 2015-09-11

**Authors:** Jiankang Han, Lei Ji, Yuehua Shen, Xiaofang Wu, Deshun Xu, Liping Chen

**Affiliations:** Huzhou Center for Disease Control and Prevention, 999 Changxing Road, Huzhou, Zhejiang 313000 China

## Abstract

**Background:**

Norovirus (NoV) has been recognized as the leading cause of both outbreaks and sporadic cases of acute gastroenteritis in children and adults worldwide. Stool samples collected from outpatients with clinical symptoms of acute gastroenteritis in all age groups at the First People’s Hospital in Huzhou, Huzhou, China between March 2014 and February 2015 were analyzed to gain insight into the epidemiology and genetic variation in NoV strains circulating in China.

**Method:**

Real-time RT-PCR (qPCR) was performed for Norovirus detection. RT-PCR were used for genomic amplification and sequencing**.** Genogroup and genotype were assigned using the NoV Noronet typing tool and the strains were named according to the time of isolation. The phylogenetic analysis was conducted using MEGA 5.

**Results:**

Of the 809 specimens, 193 (23.9 %) were positive for NoV, with GII.4 and GII.17 the most commonly identified strains. Phylogenetic analysis confirmed the presence of five recombinant strains in Huzhou. Recombinants GII.P13/GII.17 and GII.P12/GII.4 were newly detected in China. The GII.P13/GII.17 recombinant was first identified in October 2014 and steadily replaced GII.Pe/GII.4 (GII.4 Sydney 2012) as the predominant circulating NoV genotype.

**Conclusion:**

This is the first report of the detection of GII.17 in the Huzhou area and of a NoV genotype being detected in greater numbers than GII.4. Furthermore, our results indicated that following the emergence of GII.17 in October 2014, it steadily replaced the previous circulating GII.4 Sydney2012 strain, which was the dominant circulating genotype for the past 2 years. As norovirus are the important cause of nonbacterial gastroenteritis, continuous and comprehensive study of the norovirus strains involved in large and cost-effective acute gastroenteritis would help understanding the molecular epidemiology of norovirus infections and development of improved prevention and control measures.

## Background

Norovirus (NoV) is the leading cause of both outbreaks and sporadic cases of acute gastroenteritis in children and adults worldwide [1, 2]. Investigations of outbreaks have shown that the majority of transmissions are by direct contact with individuals carrying the virus, water, aerosols, contaminated food, and due to environmental contamination [3].

NoV is a member of the family Caliciviridae and has a positive-sense, single-stranded RNA genome (7.6 kb). Genetically, NoV has been classified into six genogroups (genogroup I [GI] to genogroup VI [GVI]), of which GI, GII, and GIV can infect humans [4]. The genogroups are further classified into 9 GI, 22 GII, and 2 GIV genotypes [4–6].

NoV genotype GII.4, which evolves rapidly, is associated with most NoV outbreaks and sporadic cases. New GII.4 variants emerge every 2–3 years and become the dominant strains during the new season [7]. These variants seem to have greater epidemiological fitness than other genotypes. Since 2008, at least three GII.4 variants have been circulating in the Huzhou area, China [8]. From early 2008 to late 2014, almost all outbreaks and the majority of sporadic cases in Huzhou were caused by GII.4. A new recombinant GII.P13/GII.17 was detected in sporadic cases in November 2014, and rapidly came to predominate over GII.4 in terms of number of cases. Here, we describe the emergence of GII.P13/GII.17 NoV strains and characterize the molecular epidemiology of NoV infection between February 2014 and February 2015.

## Results

### Features of NoV-associated sporadic acute gastroenteritis in Huzhou, 2014–2015

**Fig. 1:**
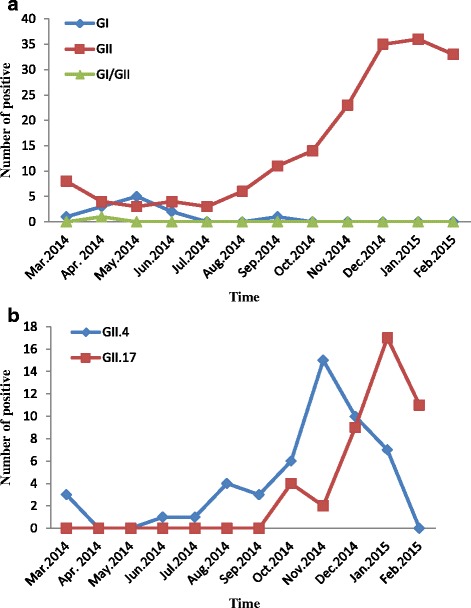
**a** Temporal distribution of NoV-positive cases in Huzhou, 2014–2015. Monthly detection of NoV in Huzhou from March 2014 to February 2015. **b** Temporal distribution of GII.4 and GII.17 in Huzhou, 2014–2015. Monthly distribution of genotype GII.4 and GII.17 in Huzhou from March 2014 to February 2015

NoVs were continuously detected throughout the year. From March 2014 to February 2015, 809 stool specimens collected from sporadic cases of acute gastroenteritis were received for NoV detection. NoV infection was found in all age groups tested (≤10y, 11–20y, 21–30y, 31–40y, 41–50y, 51–60y, and >60y). Of the 809 specimens, 193 (23.9 %) were positive for NoVs. Among these, GII NoVs comprised the greatest proportion, accounting for 93.3 % (180/193), with GII.4 and GII.17 the most commonly identified strains, GI NoVs comprised 6.2 % (12/193), and GI-GII co-infections comprised only 0.5 % (1/193). The highest NoV detection rate was during October 2014 and February 2015 (Fig. 1). However, GI NoV infection peaked from April to June 2014.

### Genotyping of NoVs

NoV-positive samples were genotyped by sequencing of regions A and C. Of the 193 norovirus positive strains, 101 were genotyped using both the capsid and polymerase genes, while 10 were genotyped using the polymerase gene alone, and 3 were genotyped using the capsid gene alone (Table 1). The most prevalent NoV genotypes were GII.Pe/GII.4 (38.6 %) and GII.P13/GII.17 (36.8 %). The GII.Pe/GII.4 genotype has been reported to predominate globally in many recent surveillance studies of NoV epidemics. The extremely high percentage of GII.17 was reported for the first time in the Huzhou area.

**Table 1:** NoV detected in the Huzhou area, China, 2014–2015

NoV genotype		Number	Percentage
GI			
ORF1/ORF2	GI.1	1	0.9
	GI.2	3	2.6
	GI.5	2	1.8
	GI.Pb/GI.6	1	0.9
	GI.7	2	1.8
ORF2^b^	GI.2	2	1.8
GII			
ORF1/ORF2	GII.Pe/GII.4	44	38.6
	GII.P13/GII.17	42	36.8
	GII.P12/GII.4	1	0.9
	GII.2	1	0.9
	GII.6	1	0.9
	GII.P7/GII.6	1	0.9
	GII.8	1	0.9
	GII.21	1	0.9
ORF1^a^	GII.P3	1	39.1
	GII.P13	6	5.3
	GII.P7	1	0.9
	GII.Pe	2	1.8
ORF2^b^	GII.17	1	0.9
Total		114	100

Distribution of genotypes according to PCR of the polymerase genes (ORF1) and capsid genes(ORF2)

^a^ORF2 not determined (negative PCR of the capsid gene)

^b^ORF1 not determined (negative PCR of the polymerase gene)

The incidence of sporadic acute gastroenteritis was higher during the 2014–2015 seasonthan previously (data not shown). An examination of the distribution of the detected NoV variants over time highlights that the emergent GII.P13/GII.17 variant steadily replaced GII.Pe/GII.4 (GII.4 Sydney 2012) as the predominant NoV in circulation in the Huzhou area (Fig. 1), which has not occurred previously. In February 2015, all positive isolates that could be genotyped were GII.P13/GII.17. This is the first report of (i) detection of GII.P13/GII.17 in the Huzhou area, China, and (ii) of a NoV genotype being detected in greater numbers than GII.4.

### Recombinant strains

Of the114 strains genotyped using two genes, 89 strainscorresponded to suspected intergenotype recombinant strains (discordant capsid and polymerase genotypes). Notable among these were GII.Pe/GII.4 (n = 44, 49.4 %), GII.P13/GII.17 (n = 42, 47.2 %), GII.P12/GII.4, GII.P7/GII.6, and GI.Pb/GI.6 (n = 1, 1.1 %). PCR of the ORF1/ORF2 junction region confirmed the recombination of these strains. Among them, recombinants GII.Pe/GII.4, GII.P7/GII.6, and GI.Pb/GI.6 have been reported previously [8]. Recombinants GII.P13/GII.17 and GII.P12/GII.4 were newly detected in China (Table 1).

### Phylogenetic analysis of GII recombinant strains

**Fig. 2:**
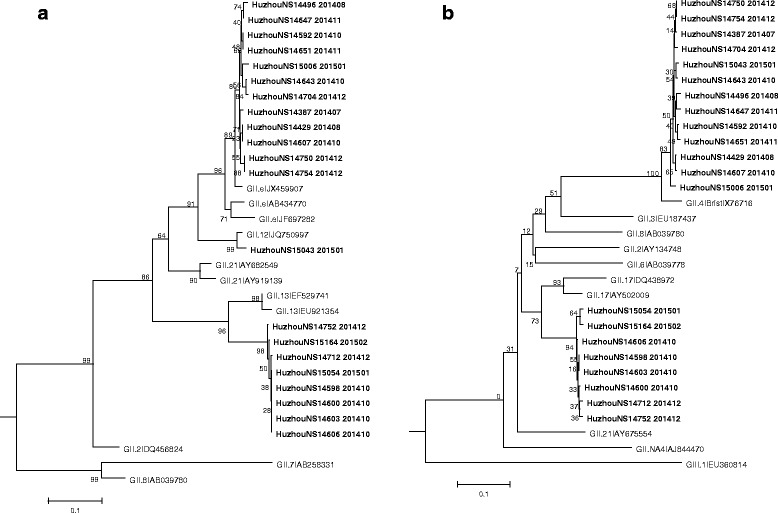
Phylogenetic analyses of recombinant GII NoV strains originating from sporadic acute gastroenteritis patients in Huzhou, 2014–2015. Phylogenetic analyses of the partial RdRp (**a**) and capsid (**b**) regions of the GIIPe/GII.4, GII.P13/GII.17, and GII.P12/GII.4 recombinants from sporadic acute gastroenteritis patients in Huzhou, 2014–2015, compared to representative strains. NoV strains detected in the Huzhou area are denoted by bold typeface

The identification of discordant genotype clustering for the partial polymerase and capsid gene sequences of NoV strains resulted in detection of two new recombinants in the Huzhou area: GII.P13/GII.17 and GII.P12/GII.4 (Fig. 2). Partial capsid gene sequence analysis of GII.17 NoVs showed that strains associated with GII.P13 polymerase segregated in a distinct cluster. This recombinant was first identified in October 2014, and thereafter rapidly became dominant type. Analysis of the GII.P12/GII.4 recombinant showed that it harbored a GII.12 polymerase gene and a GII.4 capsid gene. This strain was initially, and only, identified in January 2015. The recombinant strain GII.Pe/GII.4 is also named GII.4 Sydney 2012, which was first identified in 2012 and has been the dominant circulating strain in the Huzhou area for the past 2 years.

## Discussion

NoV is a leading cause of outbreaks and sporadic cases of gastroenteritis in individuals of all ages [9, 10] worldwide, including in the Huzhou area, China. In the present study, we analyzed NoV-associated sporadic acute gastroenteritis in the Huzhou area between March 2014 and February 2015. Our data showed that NoV circulates at high rates, and exhibits considerable genetic diversity. The circulation peak occurred during the winter–spring seasons. The circulating genotypes found in the present study differ from results obtained previously, in which GII.4 was the dominant circulating genotype, with other genotypes being detected at low frequencies [8]. Following the emergence of GII.17 in October 2014, it steadily replaced the previous circulating GII.4 Sydney2012 strain. GII.17 became the most commonly detected NoV strain in the Huzhou area by February 2015. Interestingly, young (≤10y) and old (>60y) ones are more likely to be infected with GII.4. While GII.17 prone to infect people aged from 21-50y. This is the first report of the detection of GII.17 in the Huzhou area and of a NoV genotype being detected in greater numbers than GII.4. These findings suggest that GII.17 has a selective advantage compared to GII.4 Sydney2012, possibly by accumulation of mutation in the past few years.

The periodic emergence of GII.4 NoV strains has been previously documented [11–13]. Since the late 1990s, the molecular epidemiology of NoV-associated acute gastroenteritis has been characterized by the occurrence and periodic emergence of novel variants of the NoV GII.4 lineage [12, 14, 15]. Most NoV outbreaks worldwide are caused by NoV variants of the GII.4 lineage. When a new GII.4 variant is identified it often then becomes predominant and is associated with an increase in disease activity [7, 16].

GII.4 NoV has been circulating in the Huzhou area since 2008, when monitoring of NoV was initiated. Since that time, at least three variants have been identified: 2006b, New Orleans 2009, and Sydney 2012 [8]. GII.4 2006b variants were detected in 2008 and 2009. Following the first detection of the GII.4 New Orleans 2009 variant in December 2010, they rapidly replaced GII.4 2006b and predominated from 2010 to 2011. From 2012 to the present, GII.4 Sydney 2012 accounted for the majority of both sporadic cases and outbreaks of NoV-associated acute gastroenteritis. The GII.4 Sydney-2012 variant (a recombinant of strains GII.Pe/GII.4) was first identified in Australia in March 2012. By late 2012, various countries worldwide had reported higher incidences of NoV outbreaks or illness than before, most of these were caused by the GII.4 Sydney-2012 variant [17–19]. In the Huzhou area, the GII.4 Sydney-2012 variant was first identified in November 2012, and became the predominant GII.4 variant soon thereafter. This variant caused several outbreaks in the Huzhou area between 2012 and 2014 [8].

To the best of our knowledge, there are few reports of the relative contribution of NoVs to sporadic acute gastroenteritis. Boga *et al*. [20] indicated that NoVs were less common than rotaviruses, *Campylobacter* spp., and *Salmonella* spp., but more frequent than astroviruses, adenoviruses, and *Yersinia* spp., as causes of sporadic acute gastroenteritis. Our results indicate that NoV plays an important role in sporadic acute gastroenteritis, as evidenced by the 23.9 % (193/809) positive rate. Previously, most such cases were caused by NoV GII.4, with other genotypes being detected at lower frequencies. Following the emergence of GIIP13/GII.17, GII.4 has been steadily replaced. GIIP13/GII.17 had replaced GII.4 by February 2015, becoming the predominant NoV genotype in the Huzhou area.

NoV GII.17 has been reported in children with gastroenteritis in Central and South America [21–23]), Korea [24], and Thailand [25]. Kiulia reported that NoV GII.17 predominates in selected surface water sources in Kenya [26]. GII.17 has been in existence for many years at a low frequency. However, it emerged in the Huzhou area, and now causes a greater number of cases than GII.4. It is the same condition in other part of China and also in other part of the world [27–30]. Studys show that amino acid of the new variant changed, especially in the protruding P2 domain [29, 30]. Why did GII.17 emerge suddenly? Did this genotype accumulate greater fitness, or simply undergo recombination event(s)? The answers to these questions are unknown. The biological and epidemiological success of GII.4 is, at least in part, influenced by mutations in the hypervariable P2 domain that affects the antigenic profile of the virus [31–34], which can lead to worldwide strain replacement events [18]. It is interesting to ponder whether the GIIP13/GII.17 recombinant could become the next epidemic strain like previous G11.4 strains and lead to worldwide strain replacement and an acute gastroenteritis pandemic?

The limitation of this study was the short monitoring period. Specimens were collected over a 1-year period, so whether or not GII.17 will continue to circulate is unclear. However, it is certain that GII.17 has emerged in the Huzhou area, and now outnumbers GII.4 for the first time.

## Conclusions

In summary, this is the first report of the detection of GII.17 in the Huzhou area and of a NoV genotype being detected in greater numbers than GII.4. The results showed that norovirus circulating in the Huzhou area have high diversity and new recombinants continue to emerge. Furthermore, our results indicated that following the emergence of GII.17 in October 2014, it steadily replaced the previous circulating GII.4 Sydney2012 strain, which was the dominant circulating genotype for the past 2 years. As norovirus are the important cause of nonbacterial gastroenteritis, continuous and comprehensive study of the norovirus strains involved in large and cost-effective acute gastroenteritis would help understanding the molecular epidemiology of norovirus infections and development of improved prevention and control measures.

## Methods

### Ethics statement

Institutional review board approval was not requested, as this study was part of a routine laboratory-based investigation. No human experimentation was conducted; all experiments were carried out using norovirus strains. The only human materials used were stool samples that had been sent to our laboratory for routine virological diagnosis of gastroenteritis. The results reported are associated with norovirus strains, with no patient information used.

### Specimen collection

This study was conducted at the First People’s Hospital in Huzhou, Huzhou, China as part of the regional NoV gastroenteritis surveillance program. Between March 2014 and February 2015, a total of 809 fecal samples were collected from outpatients with clinical symptoms of acute gastroenteritis. Acute gastroenteritis was defined as the presence of one or more of the following symptoms: nausea, vomiting, and/or diarrhea. Fecal samples were collected during the acute phase of infection. Acute phase means the period of time when patient have the above symptoms. All samples were obtained in a sterile container and were transported to our laboratory for immediate storage at −70 °C prior to analysis.

### Norovirus detection

Fecal samples were prepared as 10 % suspensions in phosphate-buffered saline (pH 7.4). Total nucleic acid was extracted from a 200 μL aliquot of fecal suspension using a QIAamp viral RNA mini kit (Qiagen, Hilden, Germany) according to the manufacturer’s instructions. The RNA extracts were subjected directly to reverse transcription (RT)-PCR (Polymerase Chain Reaction) or stored at −70 °C. Real-time RT-PCR (qPCR) was performed using primers and probes described previously [8]. RT-qPCR was carried out using a One Step PrimeScript RT-PCR Kit (Perfect Real Time) (TaKaRa, Dalian, China). The reaction was conducted with an initial RT step at 42 °C for 30 min, followed by 95 °C for 5 min and 40 cycles of qPCR at 95 °C for 5 s and 55 °C for 35 s.

### Genomic amplification and sequencing

Samples that tested positive for NoV by RT-qPCR were analyzed using two PCRs directed at region A of ORF1 (the 3′end of the polymerase gene ) using the primers JV12/ JV13, and at region C in ORF2 ( the 5′ end of the capsid gene ) using the primers G1SKF/G1SKR for GI and G2SKF/G2SKR for GII [35, 36]. RT-PCR was carried out using a One Step RNA PCR Kit (TaKaRa) with the following amplification conditions: RT at 50 °C for 30 min and denaturation at 95 °C for 3 min, followed by 35 cycles of 30 s at 94 °C, 30 s at 48 °C, and 30 s at 72 °C. A final extension step for 10 min at 72 °C was performed. After amplification, the PCR products were visualized by agarose gel electrophoresis. To analyze recombinant strains (capsid and polymerase of different genotypes), sequences covering the overlap between ORF1 and ORF2 were amplified by RT-PCR using primers JV12 and G1SKR/G2SKR when phylogenetic analyses indicated discordant clustering for partial sequences of the polymerase and capsid genes in the same sample. RT-PCR was carried out using a One Step RNA PCR Kit (TaKaRa). RT-PCR conditions were as follows: RT at 50 °C for 30 min and denaturation at 95 °C for 2 min, followed by 35 cycles of 30 s at 94 °C, 30 s at 48 °C, and 1 min at 72 °C. A final extension step was performed for 10 min at 72 °C. The PCR products were purified using a QIAquick PCR purification kit (Qiagen, Leusden, The Netherlands) according to the manufacturer’s instructions. The amplicons were sequenced by TaKaRa Biotechnology (Dalian).

### Genotyping and phylogenetic analysis

Genogroup and genotype were assigned using the NoV Noronet typing tool (http://www.rivm.nl/mpf/norovirus/typingtool) [6] and the strains were named according to the time of isolation. The phylogenetic analysis was conducted using MEGA 5 [37]. Phylogenetic trees with 1000 bootstrap replicates were generated using the neighbor-joining method by employing molecular evolutionary genetics analysis (MEGA), version 5.0. Reference strains were downloaded from GenBank.

### Nucleotide sequence accession numbers

The GenBank accession numbers for sequences obtained in this study are KP995319-KP995341, KR361528-KR361548.

### Availability of supporting data

The data sets supporting the results of this article is included within the article
